# Emotional Working Memory and Alzheimer's Disease

**DOI:** 10.1155/2014/207698

**Published:** 2014-02-05

**Authors:** Nicola Mammarella, Beth Fairfield

**Affiliations:** Department of Psychological Sciences, University of Chieti, Via dei Vestini 31, 66013 Chieti, Italy

## Abstract

A number of recent studies have reported that working memory does not seem to show typical age-related deficits in healthy older adults when emotional information is involved. Differently, studies about the short-term ability to encode and actively manipulate emotional information in dementia of Alzheimer's type are few and have yielded mixed results. Here, we review behavioural and neuroimaging evidence that points to a complex interaction between emotion modulation and working memory in Alzheimer's. In fact, depending on the function involved, patients may or may not show an emotional benefit in their working memory performance. In addition, this benefit is not always clearly biased (e.g., towards negative or positive information). We interpret this complex pattern of results as a consequence of the interaction between multiple factors including the severity of Alzheimer's disease, the nature of affective stimuli, and type of working memory task.

## 1. Introduction

Working memory (WM) is generally described as a memory system that temporarily maintains and manipulates different types of information during a task at hand [1] and Baddeley's [2] most recent definition of WM further highlights the role of verbal and visuospatial components and cross-modal integration during tasks that require different levels of executive processing. Moreover, WM functions have been shown to play an important role in many complex cognitive abilities, such as reading, problem solving, and spatial orientation. WM capacity, for example, reflects the ability to maintain goal-relevant information processing in the presence of competing or interfering information.

Interestingly, although studies on WM and emotion are few, recently, researchers have begun stressing the role of WM in emotional processing as well, showing how WM may be crucial for emotion regulation strategies [3]. In particular, a series of studies [4] have shown how individuals with higher WM capacity were better at suppressing emotional facial expressions and more successful at adopting an unemotional attitude while viewing emotionally charged stimuli. In addition, even though age-related difficulties are well established in classical WM tasks, many studies have repeatedly shown an age-related enhancement effect in WM tasks when emotional stimuli are involved [5, 6]. In fact, a new corpus of data seems to indicate that emotions may have a special status in WM to the extent that age-related differences may even disappear. In addition, many neuroimaging studies have highlighted less pronounced age-related changes to brain regions, for example, amygdala [7], typically involved in emotion processing. Based on this new evidence, various studies [3, 6] support the possibility that there may be an affective WM, that is, a specific WM system to maintain and manipulate affective information. Affective WM tasks are thus classical WM tasks that require the processing of affective stimuli (e.g., positive and negative pictures or words). Most interestingly, this system seems to show a different trajectory in the aging mind compared to classical WM functions and although WM deficits, in general, are typically cited as one of the principal cognitive indexes of pathological aging [8], studies about emotional effects in WM in dementia of Alzheimer's type (DAT) patients are only at the beginning.

Consequently, here, we aimed to review a series of studies with DAT patients that show emotion modulation in WM performance during the active manipulation of affective to-be-remembered stimuli. In particular, we extrapolated three major classical WM functions [1] that may be crucial for emotional processing and examined the relevant studies conducted with DAT patients.

Anticipating our conclusions, we generally found that studies using WM tasks did not provide very strong evidence for emotional enhancement effects in DAT and neuroimaging studies repeatedly showed that brain structures involved in emotion processes are disrupted in DAT. However, it may be that DAT patients do not show a general emotional enhancement effect but that they have more specific emotional effects linked to the WM function involved. After discussing the interpretation of these data, we offer suggestions about how issues of emotional processing in WM and DAT may be explored in the future, especially in terms of trying to disentagle the role of different factors that may affect emotional enhancement effects in the WM of DAT patients.

## 2. Overview

Recent research has evidenced an age-related WM resource shift towards emotional processing in Alzheimer's disease as well. In fact, there are studies that have shown preserved emotion processing in DAT patients and this efficiency of WM functions for emotional stimuli seems to be further supported by neuroimaging studies that have highlighted how the amygdala may modulate WM resources (prefrontal cortex) despite degenerative processes. These findings raise interesting questions about the integrity of WM capacity in pathological aging and this review aims at clarifying whether there may be an emotional benefit effect in DAT patients' WM.

### 2.1. Literature Search

To identify relevant studies, a comprehensive literature search in a variety of electronic databases was performed until April 12, 2013 (PubMed, PsychInfo, and Web of Science). Entered search terms were “emotion,” “Alzheimer,” “emotional processing,” “emotion regulation,” “working memory,” and “affective working memory.” In addition, reference lists from the retrieved articles were screened to identify additional papers. Articles were included for review if they met the following criteria:

1. The study sample(s) comprise(s) people with a diagnosis of dementia. Participants in the intervention studies have cognitive impairments resulting from neurodegenerative diseases, that is, a diagnosis of Alzheimer's disease. Participants fulfill either the criteria for dementia as outlined in the Diagnostic and Statistical Manual of Mental Disorder (DSM-IV-TR) or disease-specific criteria such as those for Alzheimer's dementia as formulated by the National Institute of Neurological and Communicative Diseases and the Stroke/Alzheimer's Disease. In addition, studies were included if diagnoses were based on historic information, neurologic examination, and neuropsychological assessment and supported by findings on structural and functional imaging.
2. The study used a working memory task that included emotional stimuli.
3. The actual definition of working memory shows a large variation in the literature and may include a combination of different component processes. Provided that emotional processing is involved, all studies with Alzheimer were included.
4. As for type of dementia and severity, based on Mini-Mental State Examination scores (MMSE), 23 subdivided into four categories: minimal (MMSE > 23), mild (MMSE 18–23), moderate (MMSE 10–17), and severe (MMSE < 10).
5. Experimental design.

Altogether, we reviewed 19 studies.

## 3. Working Memory Functions and Emotional Processing in DAT

Emotionally charged events are generally remembered better than neutral events even to the extent that age-related differences in WM may disappear. This pattern of performance is typically found in healthy older adults [3] and recent data investigating the encoding of emotional information in DAT patients also shows similar results. Therefore, to better clarify the interaction between emotions and WM in DAT, we present a series of studies according to the main WM function involved, maintenance, binding, and inhibition (although no single task taps just one unique WM function).

### 3.1. Maintenance 

In order for information to be processed, it must be maintained in WM, at least for a few seconds. Maintenance, therefore, is a crucial function that allows other processes to intervene and manipulate information in different ways (e.g., repetition, inhibition, binding, etc.). Typically, researchers infer short-term maintenance of information by asking participants to complete a recognition task in which old and new items are mixed and participants are asked to recognize which stimuli they had already seen or to freely recall a series of items immediately after their presentation.

Schultz et al. [9] tested immediate recall for valenced pictures and found a general emotional enhancement effect in DAT patients. On the contrary, a study by Satler and Tomaz [10] showed better performance with negative trials compared to positive or neutral trials. This finding is in line with the negativity bias previously found in a study by Döhnel and colleagues [11] with mild cognitive impairment (MCI) patients. This negativity bias was also found in another study by Fleming and colleagues [12] which showed better immediate free recall for negative words in DAT patients and in the study by Boller et al. [13] in which DAT patients recalled negative stories better. Negative emotions, therefore, seem to be maintained in WM and remembered better by DAT patients compared to positive and neutral ones.

### 3.2. Binding

When we perceive the world around us, we must first simultaneously process separate features and subsequently bind them together in order to form unique and memorable objects, scenes, and/or episodes. Above all, this function is crucial in all cognitive tasks that require integration of information coming from different types of material (e.g., verbal and visuospatial), modalities (e.g., visual and acoustic), and domains (cognitive versus emotional). Typically this function is studied by asking participants to remember a series of objects or pictures in their studied locations. For example, Huijbers and colleagues [14] used a picture recollection task in which their participants studied a grid where 9 pictures appeared sequentially. At the end of each trial, participants were required to replace each picture in its exact location. In this case, DAT patients performed better on negative trials compared to neutral ones, as did the control group. In a similar binding task, Borg et al. [15] asked participants to recognize the correct location of valenced pictures. Results showed how DAT patients remembered the locations of the positive pictures better than the locations of the negative and neutral pictures. On the contrary, Nashiro and Mather [16] did not find any valence effects on DAT performance when they controlled for arousal and not only valence. Data on emotions and binding functions therefore are ambiguous and binding functions seem to be influenced in different ways according to the type of stimuli and task used.

### 3.3. Inhibition

Inhibition in WM involves paying attention to only some of the to-be-processed information. Selection and inhibition allow only relevant information to be further manipulated in WM. Generally speaking, inhibition refers to an individual's control over the access of relevant and irrelevant information and has been studied with different complex tasks (e.g., Stroop test and Daneman & Carpenter's Reading Span, to cite only a few). In a typical WM Span Task [17, 18], participants are required to process (read and judge) a series of sentences, words, or operations and remember final words/digits in their correct serial order. These tasks have been shown to entail concurrent processing and short-term storage demands coupled with an attentional control component necessary for limiting interference between processing and storage. Mammarella et al. [19] assessed age-related differences in healthy older adults and DAT patients using a modified version of the operation WM Span Test [20] that included positive and negative words as well as neutral words (as in the classical working memory task). Participants judged simple math operations during the processing phase and then had to actively maintain a set of unrelated words that were either neutral, as in the original task, positive or negative in memory. Finally participants were asked to remember all of the final target words (affective and neutral ones).

Results showed that while healthy older adults performed better than DAT patients with valenced words, DAT patients did not show any emotional benefit.

Differently, Doniger and Bylsma [21] found that moderate DAT patients showed larger interference effects with positive and negative trials in an emotional version of the Stroop task. These results seem to indicate that suppressing the emotional valence of items is more difficult and suggest that DAT performance was influenced by emotion.

### 3.4. Summary

On one hand, the complex evidence presented above is consistent with the idea that emotional information has a preferential access to WM even in DAT patients. This preferential access means having more opportunities for WM to process emotional information. This assumption is based on the idea that the emotional effect in WM, although linked to more automatic and early attentional responses (e.g., fast detection of emotional stimuli) may rely on WM representations associated with emotional personal events.

On the other hand, it also highlights that the processing of emotional information in WM may fail due to the fact that additional control is required in order to orient attention towards this aspect and prevent distraction. Moreover, it may also indicate that emotional valence effects do not occur early in the processing stage but rather arise later during the maintenance and use of emotional information when a greater amount of cognitive resources is required. In line with this, only valenced information that does not recruit a greater amount of cognitive resources will be favoured (e.g., negative information). In fact, studies on healthy aging [22] have shown that the tendency to remember positive information better than negative information is based on recruitment of control processes.

Consequently, DAT patients may show different effects according to emotional valence.

## 4. Working Memory, Emotion Regulation, and DAT

The ability to manipulate valenced information in WM is a critical component of emotion regulation processes. For example, negative and positive stimuli elaboration may be attenuated or amplified in a way that it may increase/decrease people's vulnerability to mood disorders [23]. Thus, the interaction between valence and WM functions may have direct effects on emotion regulation abilities. Henry et al. [24] conducted one of the first studies that tested the ability of DAT patients to apply different emotion regulation strategies online. In particular, they asked a group of healthy older adults and a group of DAT patients to suppress (e.g., hide their feelings as much as they can) or amplify (e.g., show their feelings as much as they can) their feelings while watching a film clip. Results showed that behavioral amplification of expressed emotion was disrupted in DAT, while the ability to inhibit the expression of ongoing emotions was relatively preserved. Similarly and more recently, Goodkind and colleagues [25] showed that when DAT patients were told when the aversive event would occur but not given instructions about downregulation, they were able to spontaneously suppress emotional facial behavior just as well as normal controls. This small set of data points to the idea that the difficulty found in amplifying emotions might reflect decreasing controlled processes in DAT such as WM resources, while relatively intact suppression of emotions could reflect more reliance on automatic inhibitory mechanisms. Reduced WM functions may thus be related to the observed difficulties in emotion upregulating processes in DAT. One explanation may be the fact that prefrontal regions show greater regulation-related activation and the functional efficacy of those structures depends on underlying cognitive ability [26].

### 4.1. Summary

Although several aspects of emotion seem to be intact in DAT patients, emerging evidence shows that patients have an impaired ability to adaptively regulate their emotions. In addition, evidence regarding emotion regulation processes and their relationship with WM in DAT is scarce. Undoubtedly part of the reason for this is that it is difficult to study emotion regulation strategies such as cognitive reappraisal in patients with cognitive difficulties. In fact, depending on task demands, measures of emotion regolation invariably involve additional skills such as face perception, expressive speech, and/or semantic knowledge pertaining to an emotion label, all of which are impaired in DAT patients. What is clearly needed, similar to what has been developed for WM tasks targeting cognition, is an effort to develop new WM tasks based on the use of different automatic emotion regulation strategies.

## 5. Neural Correlates of WM Emotional Processing in DAT

Neuroimaging studies have explained the emotional enhancement effect in long-term memory in terms of an interaction between the amygdala and brain regions that are involved during encoding and retrieval of emotional events [27]. Specifically, memory for emotional events relies on a fronto-amygdala-hippocampal circuit with the amydala modulating prefrontal cortex activity (orbital and dorsolateral) and the hippocampus. fMRI studies have evidenced amygdala activity together with activation in frontoparietal regions during WM tasks with emotional stimuli as well [7]. In particular, Döhnel et al. [11] found greater activation in prefrontal and parietal regions during an *n*-back WM task with emotional faces associated with a greater amygdala activation. These data have been explained in terms of an interaction between the amygdala and the orbitofrontal cortex which plays a crucial role in attributing emotional qualities to stimuli. This information is subsequently maintained and manipulated in the dorsolateral prefrontal cortex (PFC) [28]. Given the well established role of dorsolateral PFC in WM functions [29], it is likely that emotional events are remembered better because they capture a greater amount of perceptual and attentional resources (as the contemporary activation of visual brain regions may indicate). In particular, with regards to aging studies, neuroimaging evidence shows that the brain regions and circuits involved in emotion processing are less sensitive to aging when compared to other brain regions [6]. In addition, when Mather and colleagues [30] asked a group of younger (mean age 23) and a group of older (mean age 78) adults to rate a series of positive, negative, and neutral pictures on a 7-point scale in terms of arousal (from calm to excitement), fMRI data show similar levels of amygdala activation across the two groups and a greater activation for positive pictures in the older adults group alone. Modulating functions of the amygdala are, therefore, well preserved during aging. Findings with older adults do not seem to reflect only early activation following automatic limbic responses. They also reflect later involvement of neural circuits that allow emotional stimuli to be maintained in WM and bound to autobiographical events. For example, Kensinger and Schacter [31] detected greater activation in the medial PFC and the cingulate gyrus in a group of older adults compared to younger adults. These regions are known to be typically involved in autoreferential processing, highlighting the tendency for older adults to link online information to personal events or to the generation of regulation strategies.

Studies of emotional long-term memory in DAT [32] have repeatedly explained the absence/presence of emotional enhancement effects in Alzheimer's disease as due to the specific stage of atrophy in amygdala and hippocampal structures. For example, Horinek et al. [33] found the same degree of hippocampal and amygdalar volume loss. This data is in line with previous studies [7] which found the same rate of degeneration in both structures. Poulin et al. [34] also found that amygdala atrophy is comparable to that of the hippocampus from the earlier stages of dementia on. In detail, with regards to WM functions, Perrin et al. [35] found that AD patients' picture recall did not benefit from positive emotional context (a positive sound associated to the picture) and again explained this data in terms of WM limitations due to amygdala atrophy and early frontotemporal dysfunctions. More interesting is the PET study by Rosenbaum et al. [36] which found preserved influence from the left amygdala and left hippocampus on left and then right inferior PFC, in the absence of a direct amygdala-hippocampus influence using a match-to-sample face recognition task. This data seems to indicate that DAT patients may still use some emotional brain circuits and show an emotional enhancement effect in WM despite their well-known amygdala and hippocampus deficits.

### 5.1. Summary

In summary, since memory for emotional stimuli involves a variety of encoding and retrieval processes, it seems reasonable to assume that different brain regions may be involved at different moments. Generally speaking, neuroimaging studies have shown that the prefrontal cortex supports short-term maintenance and manipulation of emotional stimuli in WM and that the amygdala modulates the activity in these brain regions during the different stages of emotion processing. This circuit seems to be less sensitive to pathological aging processes compared to other brain regions.

## 6. Recommendations for Future Research

The complex picture of emotional effects in WM of DAT leads us to several considerations. First, it is important to further our knowledge about the relationship between emotion and WM measures. In fact, WM tasks are generally grouped in recall-based more “active” tasks such as the classical Daneman and Carpenter's Reading/Listening Span [17], the Operation Span [18], the active visuospatial task [37], and recognition-based “more passive” tasks such as *n*-back, recency-probe paradigms, matching-to-sample, and binding tasks to cite only a few [38]. All of these tasks, however, differ in the type and the amount of processing required as well as in the nature of the information to be temporarily maintained.

An interesting attempt to reconcile different pattern of results with emotion, WM, and DAT would be to use different tasks and highlight the role of specific WM functions involved and specialized for affective processing. In fact, the majority of studies did not investigate the ability of actively maintaining and storing information to achieve the task goals as classical WM tasks require.

Second, the majority of studies with DAT did not carefully control for different affective dimensions of study material. For example, some studies [10] used high- versus medium-arousal valenced pictures, while others [16] used arousing versus nonarousing items. Still others [9] did not mention whether arousal was controlled. In sum, it is not clear whether emotional enhancement effects in DAT patients are mainly due to arousal effects, as suggested by Nashiro and Mather [16], valence effects, or both. From the WM studies, it is likely that WM for high arousing and negative valenced stimuli is preserved in DAT, but we need further studies to assess the role valence in WM modulation.

Third, group characteristics may be called on to explain discrepancies across studies. In fact, differences among the samples of DAT patients may also explain the mixed findings of whether DAT patients demonstrated an emotional benefit in their WM performance. These differences may be linked to different stages of deterioration in the brain regions involved in emotion and cognition processing.

Finally, the majority of studies reviewed here used affective visuospatial material. A long-term memory study by Kensinger and colleagues [39] that used verbal material, in fact, did not detect any emotional benefit. This is in line with Mammarella et al.'s study [19] that used a verbal WM task and did not detect an emotional effect with DAT patients. Thus, differences in the type of affective material may be relevant as well. Pictures, in fact, may create richer memory traces that may generate larger emotional enhancement effect not detectable with affective words.

A concluding thought must go to a motivational explanation of this complex picture of data as motivation towards emotional goals has been shown to be crucial to understanding the trajectory of the aging mind towards Alzheimer's disease [40]. According to this approach, lack of motivation is considered as one of the main characteristics of dementia. Given that motivation towards meaningful emotional goals needs recruitment of cognitive resources towards emotion processing [41], results typically obtained in WM emotional tasks with DAT may depend on the lack of attentional and WM processes motivated towards emotion processing. Importantly, this hypothesis may highlight how different levels of motivation towards emotional goals may lead to different degree of emotional effects in WM also in DAT.

## 7. Conclusions

This review highlights the relevance of WM emotional processing studies in individuals with DAT. These emotional gains are mostly confined to maintenance functions of WM. However, general positive effects may be obtained in the early stages of dementia across a variety of WM functions. Undeniably, people with dementia can still use emotional stimuli and this may have relevant daily life implications. Studies conducted in our lab show that emotional WM tasks can best be used with DAT provided that the experimenter models each task step and provides verbal cues to guide the patient. Correct verbal instructions and asking patients not to guess are very important steps for the outcomes of an emotional WM task. Emotional WM integrity may help build up general activity levels, motivation necessary for undertaking new activities, and the sense of competence, which together may result in better quality of life for people with dementia. Finally, the concept of emotional WM may provide health professionals with an opportunity to interact with their patients in a more effective manner, focusing on residual emotional abilities and learning capacities rather than deficits and decline. We hope that the evidence reviewed in this study fosters both emotional WM researches and gives new insights to improve emotion-based clinical practice with DAT.
